# Rainfall can explain adaptive phenotypic variation with high gene flow in the New Holland Honeyeater (*Phylidonyris novaehollandiae*)

**DOI:** 10.1002/ece3.352

**Published:** 2012-08-28

**Authors:** Steven A Myers, Stephen Donnellan, Sonia Kleindorfer

**Affiliations:** 1 School of Biological Sciences, Flinders UniversityBedford Park, Adelaide, 5001, Australia; 2 South Australian MuseumNorth Terrace, Adelaide, 5000, Australia; 3 Australian Centre for Evolutionary Biology and Biodiversity, University of AdelaideAdelaide, 5005, Australia

**Keywords:** Adaptation, birds, natural selection and contemporary evolution, population ecology, population genetics – empirical

## Abstract

Identifying environmentally driven changes in traits that serve an ecological function is essential for predicting evolutionary outcomes of climate change. We examined population genetic structure, sex-specific dispersal patterns, and morphology in relation to rainfall patterns across an island and three peninsulas in South Australia. The study system was the New Holland Honeyeater (*Phylidonyris novaehollandiae*), a nectarivorous passerine that is a key pollinator species. We predicted that rainfall-related mechanisms would be driving local adaptation of morphological traits, such that in areas of lower rainfall, where nectar is less available, more insectivorous traits – shorter, deeper bills, longer tarsi, and longer wings – would be favored. The study populations differed in phenotype across the Eyre, Yorke, and Fleurieu Peninsulas and Kangaroo Island despite high gene flow (single continuous population) and sex-biased dispersal (males were philopatric and females dispersed). We tested the role of rainfall in shaping the observed phenotypic differences, and found strong support for our predicted relationships: birds in areas of higher rainfall had higher condition indices, as well as longer bill-head length, deeper bills, and shorter tarsi. Bill depth in males in high-rainfall sites showed signals of stabilizing selection, suggesting local adaptation. In addition to these local indications of selection, a global pattern of directional selection toward larger size for bill-head length, bill-nostril length, and wing length was also observed. We suggest this pattern may reflect an adaptive response to the relatively dry conditions that South Australia has experienced over the last decade. We conclude that rainfall has shaped aspects of phenology in *P. novaehollandiae*, both locally, with different patterns of stabilizing and directional selection, and globally, with evidence of adaptive divergence at a landscape scale.

## Introduction

Climate change has a worldwide impact upon biodiversity and is considered responsible for rapid changes in the phenology and distribution of many plant and animal species (Root et al. 2003; Thomas et al. 2004; Parmesan 2006; Peterson et al. 2008; Olsson et al. 2011). Populations can respond to conditions of changing climate by either (1) emigrating to avoid the change, or (2) remaining and becoming exposed to the change. Adaptation is likely to follow in populations exposed to climate change, either via processes of plasticity or natural selection; if selection pressure is high enough, then local extinction may follow (Davis and Shaw 2001; Charmantier et al. 2008; Hannah et al. 2008; Raxworthy et al. 2008; Chevin et al. 2010). Given the acceleration of climate change through anthropogenic activity, research now focuses on the adaptive capacity of organisms to respond to rapid climate change (Allen et al. 2000; Rosenzweig et al. 2008) to help understand the current and likely future consequences of climate change (Deutsch et al. 2008; Huntley et al. 2008; Visser 2008; Kearney et al. 2009). One way to monitor animal response to climate change is to measure the strength of selection and rate of evolution of functionally important traits in the context of environmental variation (Gienapp et al. 2008). Identifying environmentally driven changes in traits that serve an ecological function will increase our theoretical understanding of the timeframe for adaptive response to climate, and will inform the conservation management of species with key ecosystem functions. Birds are an ideal model system to examine selection because many facets of their foraging ecology and survival can be predicted from specific morphological traits (Bowman 1961; Lederer 1975; Fitzpatrick 1985; Blomqvist et al. 1997; Keast and Recher 1997; Grant 1999; Forstmeier and Keßler 2001). Here, we investigate the role of selection and genetic drift on divergence in functional morphological traits in a key pollinator species, the New Holland Honeyeater (*Phylidonyris novaehollandiae*), across an environmentally variable landscape in southern South Australia.

The majority of ornithological studies have been conducted in the Northern Hemisphere where avian life history strategies differ discernibly from those in the Southern Hemisphere (see Martin 1996). Long distance migration is a common trait shared by Northern Hemisphere birds that is likely to confound investigation of a species' adaptive response to local environmental change, as it creates two strong and potentially contrasting periods of selective pressure per year (that is, non-breeding and breeding seasons). In contrast, most Australian songbirds are opportunistically nomadic, remaining in or near breeding territories and tracking local resources as they become available (Collins et al. 1984; Higgins and Peter 2002). We use the New Holland Honeyeater to examine the role of local environmental conditions on variation in morphology for two main reasons: (1) *P. novaehollandiae* follows local nectar resources as they become available, indicating strong reliance on food resources that are influenced by environmental conditions (McFarland 2002); and (2) movement records (*n* = 11,260) show that long distance dispersal is not common, with 99.1% of birds moving <10 km from location of banding (Higgins and Peter 2002), suggesting that populations endure local environmental conditions year-round. This relatively contained movement coupled with a specialist diet makes *P. novaehollandiae* an ideal model for the investigation of adaptive responses to environmental variation.

The diet of *P. novaehollandiae* has two major components: nectar and aerial insects (Ford and Paton 1977; Recher 1977; Paton 1982). Nectar (and other foods rich in carbohydrates such as manna, honeydew, and lerp) takes preference as the main source of energy for *P. novaehollandiae*, while insects are primarily a protein supplement (Paton 19820; Paton 1982; Ford and Paton 1982; Collins et al. 1984). *P. novaehollandiae* spend the majority of their time foraging for carbohydrates and are most likely to be limited by their ability to meet their energy requirements from carbohydrates than by their ability to meet their protein requirements (Paton 1982). Nectar production, and hence availability, is directly influenced by rainfall (Porter 1978; Armstrong 1991a,b; Wyatt et al. 1992; Wooller et al. 1998; Keasar et al. 2008; Watson 2011), with periods of high rainfall resulting in increased nectar productivity; therefore, nectar should be more available in wetter climates. South Australia is characterized by spatially variable rainfall with arid, semi-arid, and dry sub-humid climates (Australian Bureau of Meteorology; http://www.bom.gov.au/index.shtml), allowing for comparison between sites experiencing high- and low-rainfall conditions. The climate system in South Australia is sometimes referred to as having unpredictable “boom and bust” conditions, namely high- or low-rainfall periods, respectively (Kingsford et al. 1999; Jenkins et al. 2005; Robin et al. 2009).

Evidence for an increasingly insectivorous diet under conditions of reduced nectar availability, including divergence of key functional morphological traits and foraging behavior, has been observed previously in *P. novaehollandiae* (Myers et al. 2010). Therefore, under conditions of lower rainfall, we predict reduced nectar availability, which should drive niche expansion toward an increasingly insectivorous diet. This niche expansion should be reflected by changes in morphological traits that have a key role in feeding and foraging ecology, including bill shape, wing length, and tarsometatarsal length. Shorter, deeper bills provide increased crushing force and allow handling of larger prey items (Bowman 1961; Lederer 1975; Grant 1999). Typically, more insectivorous honeyeaters have shorter, less slender bills than nectarivorous birds (Lederer 1975; Wooller 1984; Wooller and Richardson 1988). Wings with larger area allow increased aerial agility that is important in sallying (Warrick et al. 2002), while longer tarsi improve the efficiency of gleaning behavior during foraging (Fitzpatrick 1985) and increase spring, allowing quicker sallying from a perch (Sherry 1982). Gleaning and sallying are typical insect foraging behaviors in *P. novaehollandiae* (Myers et al. 2010); therefore, longer wings (assuming wing length is a reliable indicator of wing area) and tarsi are expected to be characteristic of a more insectivorous honeyeater. Having said this, an increased reliance on alternate carbohydrates such as manna and honeydew in *P. novaehollandiae* may occur during low-rainfall periods (Paton 1982), and we are mindful of the potential influence this may have on morphology – shorter bills and longer tarsi are expected to be favored during drier conditions, coinciding with the expected shift based on increasing insectivory. Therefore, we predict less nectar consumption by *P. novaehollandiae* in the parts of their range with lower rainfall, and a subsequent shift toward shorter, less slender bills, longer wings, and longer tarsi. In summary, we predict phenotypic divergence across a climatically variable landscape, driven by local adaption to prevailing conditions.

In this study, we have two main aims: (1) to examine population genetic structure and sex-specific dispersal, and (2) to examine variation in morphological foraging traits in relation to rainfall. For the study of morphology in relation to rainfall, we (3) examine morphological variation in *P. novaehollandiae* across a landscape in which rainfall is spatially variable; (4) test the role of genetic drift on morphological variation; and (5) test for signals of selection on morphological traits, using body condition as a proxy for fitness.

## Materials and Methods

### Study sites

**Figure 1:**
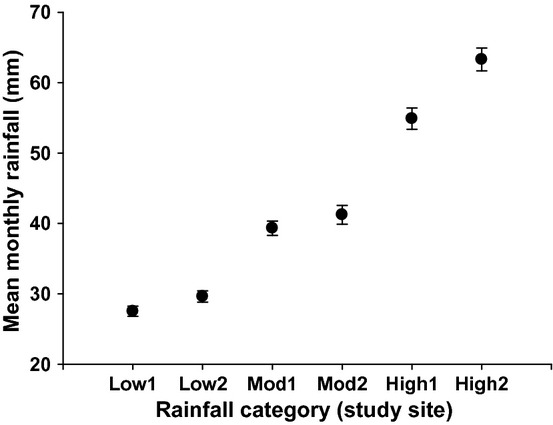
Monthly rainfall (mean ± SE; mm) at each study site during the study period (2005–2009).

**Figure 2:**
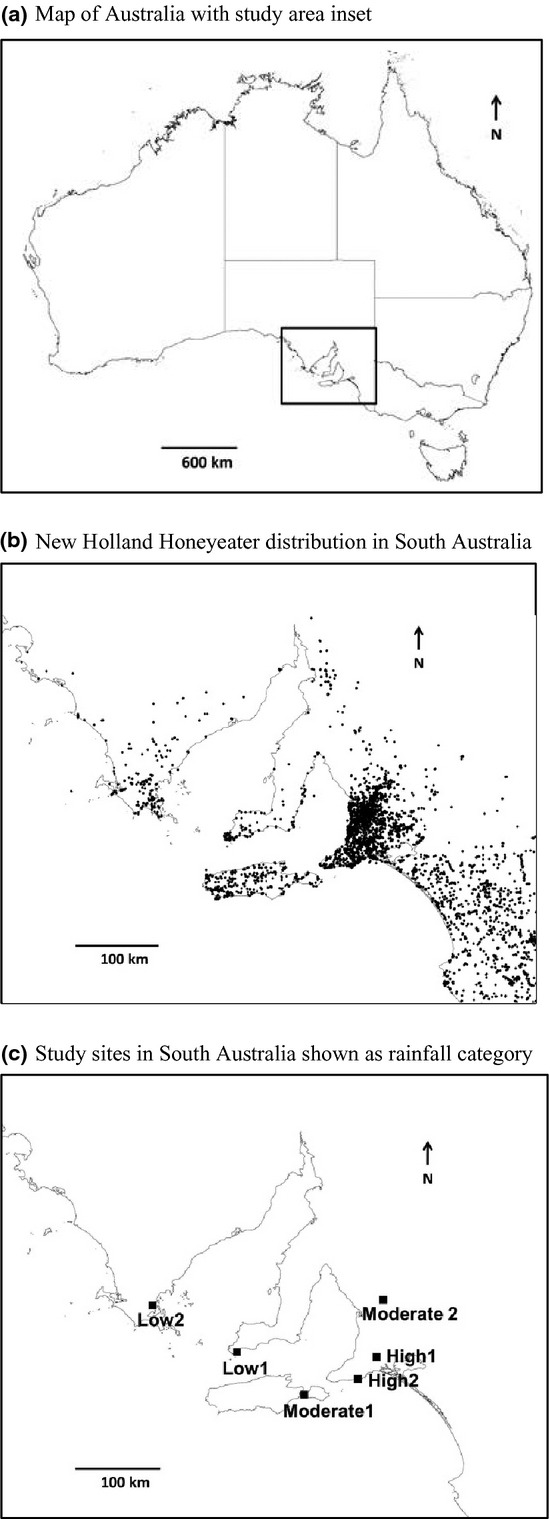
Maps showing (a) Australia with study area inset; (b) New Holland Honeyeater distribution for South Australia based on museum vouchers and sight records from the South Australian Department for the Environment and Heritage; and (c) study sites in South Australia with the rainfall categories per study site (Low, Moderate, High). [Correction added on 12 September 2012, after first online publication: For Fig. 2(c), the site designations “Low1” and “Low2” have been reversed].

We studied *P. novaehollandiae* at six sites in southern South Australia. Each site represented one of three rainfall categories: low (<32 mm), moderate (32–44 mm), and high (>44 mm) (Fig. 1) mean monthly rainfall (measured from within the study period); and each category was represented by two replicate sites: Low Rainfall 1 (Innes Conservation Park); Low Rainfall 2 (Port Lincoln); Moderate Rainfall 1 (Pelican Lagoon Research Station); Moderate Rainfall 2 (Sandy Creek Conservation Park); High Rainfall 1 (Scott Conservation Park); High Rainfall 2 (Newland Head Conservation Park) (Fig. 2c). Vegetation at each site, excluding “Low Rainfall 2,” is described in Rix (1976), Ford and Paton (1977, 1982), Westphal et al. (2003), Kleindorfer et al. (2006), Schlotfeldt and Kleindorfer (2006), Galligan and Kleindorfer (2008), Oorebeek et al. (2009), Myers et al. (2010). Vegetation at “Low Rainfall 2” (Port Lincoln) comprises mallee: Coastal White Mallee (*Eucalyptus diversifolia*) and Yorrell (*Eucalyptus gracilis*) are dominant tree species found throughout thickly vegetated areas; *E. diversifolia* is often accompanied by understorey species including Wallowa (*Acacia calamifolia*), Coastal Velvet-bush (*Lasiopetalum discolour*), Felted Wallaby Bush (*Beyeria lechnaultii*), Dwarf Hop Bush (*Dodonaea humilis*), and Shiny Ground Berry (*Acrotriche patula*).

### Rainfall

For each site, we calculated mean monthly rainfall from across the study period using data from 4 months when survival and/or reproductive success in *P. novaehollandiae* are (is) predicted to be most influenced by rainfall: 2 months toward the end of the dry season (March, April), when nectar is expected to be least abundant, and 2 months during the wet season (August, October), when nectar is expected to be most abundant. Rainfall data were obtained from the Australian Bureau of Meteorology (http://www.bom.gov.au/index.shtml) using the nearest meteorological station to each site (minimum distance = 0 km; maximum distance = 51 km; average distance = 17 km): “Low Rainfall 1” (Innes National Park): Warooka meteorological station; “Low Rainfall 2” (Port Lincoln): North Shields meteorological station; “Moderate Rainfall 1” (Pelican Lagoon Conservation Park): Cape Willoughby meteorological station; “Moderate Rainfall 2” (Sandy Creek Conservation Park): Rosedale meteorological station; “High Rainfall 1” (Cox Scrub/Scott Conservation Parks): Kuitpo Forest Reserve meteorological station; and “High Rainfall 2” (Newland Head Conservation Park): Parawa meteorological station.

### Sample collection

We used only adult birds, aged by morphology (Disney 1966; Higgins and Peter 2002). We mist-netted a total of 503 birds between 2005 and 2009 at the six sites. We sampled across 5 days at each site twice per year: once during the breeding season and once during the non-breeding season, corresponding with the months when *P. novaehollandiae* should be most responsive to rainfall stimuli. Each bird was banded with an aluminum identification band, measured for morphological characteristics, and sampled for blood that was stored on FTA paper for DNA analysis (see Kleindorfer et al. 2006).

### Sex determination

A subset of birds (*N* = 432) were sexed using the molecular genetic method of Kahn et al. (1998), using conditions outlined in Jensen et al. (2003). DNA was extracted from FTA using method #4 of the protocols outlined by Smith and Burgoyne (2004). The remaining birds (*N* = 71) had sex assigned using a univariate morphology-based sexing method (Myers et al. 2012) that allowed for regional variation in morphology (Ellrich et al. 2010).

### Morphology

At the time of banding, we measured seven morphological traits: (1) bill length from the tip of the bill to the back of the head (bill-head length); (2) bill length from the tip of the bill to the anterior extreme of the nostril (bill-nostril length); (3) bill depth measured at the base of the bill (bill depth), (4) bill width measured at the base of the bill (bill width); (5) length of the flattened wing (wing length); (6) tarsometatarsal length (tarsus length); and (7) body mass (mass). All traits, excluding mass, were measured to the nearest tenth of a millimeter using callipers. Mass was measured to the nearest tenth of a gram using scientific scales. All measurements were made by SK (*N* = 386) and SM (*N* = 117), who measured birds in all six study sites. Variation in measurement between researchers for all traits was not significant (*t*-test; *P* > 0.05), and was less than the variation across sites. A second test of measurement error tested morphological variation across sites for each researcher separately, which mirrored the findings for both researchers combined, indicating a low degree of measurement error.

Multivariate analysis of variance (MANOVA) with sex as a fixed factor, as well as study site and year, showed that morphological variation differed significantly between the sexes (*F* = 38.51; *P* < 0.001; Wilk's Lambda = 0.66; Partial ETA2 = 0.34). No significant interaction effect was observed between sex and either study site or year, indicating that morphological variation between sexes did not change across study sites or years. Examination of trait means for each sex indicated that males had larger measurements for all traits (Table 1), consistent with previous observations of sexual dimorphism in *P. novaehollandiae* (Disney 1966; Rogers et al. 1986; Higgins and Peter 2002; Myers et al. 2012). Due to this variability between sexes, and anticipated variation in life histories (Greenwood 1980; Clarke et al. 1997), we chose to investigate the sexes separately, where possible, for all further analyses. We used MANOVA (SPSS 19.0; IBM® SPSS® Statistics, Armonk, NY) with study site as a fixed factor to test if *P. novaehollandiae* showed significant morphological variation across study sites. To further explore the potential influence of rainfall on morphological variation, we used multiple regression, with rainfall as the dependent variable, and the morphological traits as the independent variables. If rainfall is influencing morphological variation as we predicted, then a correlation that reflects the predicted interactions between rainfall and morphology should be observed.

**Table 1:** Morphological variables (mean ± SD) in New Holland Honeyeaters across study sites that differ in annual rainfall (see Methods) in South Australia for (a) males and (b) females. The rainfall categories and study sites are as follows: Low Rainfall 1 (Innes National Park; Yorke Peninsula); Low Rainfall 2 (Port Lincoln; Eyre Peninsula); Moderate Rainfall 1 (Pelican Lagoon Conservation Park; Kangaroo Island); Moderate Rainfall 2 (Sandy Creek Conservation Park; Fleurieu Peninsula); High Rainfall 1 (Cox Scrub/Scott Conservation Parks; Fleurieu Peninsula); High Rainfall 2 (Newland Head Conservation Park; Fleurieu Peninsula)

(a) Male morphology						
	Low rainfall 1 (*N* = 91)	Low rainfall 2 (*N* = 35)	Moderate rainfall 1 (*N* = 37)	Moderate rainfall 2 (*N* = 70)	High rainfall 1 (*N* = 60)	High rainfall 2 (*N* = 80)
Bill-head length	41.0 ± 0.9	40.0 ± 1.3	42.5 ± 0.9	42.4 ± 0.8	41.7 ± 1.2	42.2 ± 0.9
Bill-nostril length	10.2 ± 0.5	9.6 ± 0.7	11.0 ± 0.9	10.9 ± 0.6	10.4 ± 0.6	10.5 ± 0.7
Bill depth	5.3 ± 0.4	5.3 ± 0.4	5.1 ± 0.3	5.2 ± 0.3	5.1 ± 0.3	5.1 ± 0.2
Bill width	5.3 ± 0.4	5.3 ± 0.6	5.2 ± 0.4	5.2 ± 0.4	5.2 ± 0.4	5.2 ± 0.3
Wing length	77.7 ± 2.3	77.1 ± 3.0	78.8 ± 2.7	79.8 ± 2.6	77.0 ± 3.3	78.9 ± 2.4
Tarsus length	23.6 ± 0.9	23.5 ± 0.9	23.7 ± 0.8	23.6 ± 0.9	23.0 ± 0.9	23.0 ± 0.8
Mass	20.2 ± 1.1	20.1 ± 1.8	21.2 ± 1.8	22.3 ± 1.9	21.0 ± 1.4	22.2 ± 1.8

(b) Female morphology						
	Low rainfall 1 (*N* = 43)	Low rainfall 2 (*N* = 24)	Moderate rainfall 1 (*N* = 35)	Moderate rainfall 2 (*N* = 36)	High rainfall 1 (*N* = 35)	High rainfall 2 (*N* = 66)
Bill-head length	38.6 ± 1.1	38.3 ± 0.9	39.7 ± 0.8	39.7 ± 0.9	40.00 ± 1.2	40.0 ± 1.0
Bill-nostril length	9.6 ± 0.6	9.5 ± 0.6	10.1 ± 0.8	9.9 ± 0.6	9.9 ± 0.7	9.9 ± 0.5
Bill depth	4.9 ± 0.3	5.0 ± 0.3	4.9 ± 0.3	5.0 ± 0.3	4.8 ± 0.3	4.8 ± 0.3
Bill width	4.9 ± 0.3	4.9 ± 0.3	4.8 ± 0.4	5.1 ± 0.4	4.9 ± 0.4	4.9 ± 0.3
Wing length	71.8 ± 2.8	72.3 ± 3.4	73.8 ± 2.1	73.9 ± 2.7	74.2 ± 3.0	73.9 ± 2.7
Tarsus length	22.4 ± 1.0	22.5 ± 0.8	22.6 ± 0.6	22.5 ± 0.8	22.4 ± 0.8	22.4 ± 0.7
Mass	18.0 ± 1.5	18.4 ± 1.2	19.1 ± 1.4	19.7 ± 1.9	19.9 ± 1.9	19.5 ± 1.8

### Body condition in relation to morphology and rainfall

Many of our predictions in this study rely on the assumption that conditions for survival and/or reproduction are more favorable in areas of higher rainfall. We tested this assumption indirectly by comparing the mean of a body condition index (CI), calculated as the standardized residuals of an ordinary least-squares linear regression of mass against tarsus length (Jakob et al. 1996; Merilä et al. 2001b), and mean monthly rainfall across our study sites using linear regression analysis. Our test was one-sided, testing the hypothesis that condition and rainfall were positively correlated in a linear fashion.

Natural selection typically drives population phenotypes toward fitness peaks. This relationship may be used to identify signals of selection, including the mode and direction of selection (Merilä et al. 2001a,b). We used condition index as a proxy for individual fitness (Gillespie 1998; Moya-Laraño et al. 2008). We tested for signals of selection by comparing condition index between individuals with morphological trait values within 1 standard deviation of the mean (mean trait value), values between 1 and 2 SD smaller than the mean (extremely small trait values), and values between 1 and 2 SD larger than the mean (extremely large trait values). We did this for each morphological trait (with the exception of tarsus length, as it was used in calculating the condition index), within each rainfall category, for each sex using one-way between-groups analysis of variance. The relationships observed between condition index and morphology were examined graphically. If stabilizing selection is driving morphological variation, as we predicted, then the condition index should be highest in individuals with mean trait values, and this pattern should be repeated across rainfall categories. However, if disruptive selection is driving morphological variation, the condition index should be highest in birds with trait values at both extremes; and if directional selection is driving morphological variation, the condition index should be highest in birds with trait values at a single extreme, or at the mean and a single extreme.

### Molecular genetic analysis

We genotyped 330 individuals at 10 microsatellite loci: *Pn2, Pn3, Pn4, Pn5, Pn8, Pn13, Pn15, Pn22, Pn23, and Pn25* (Myers et al. 2009). Polymerase chain reaction conditions were as outlined by Myers et al. (2009). Prior to performing analyses, we tested the suitability of our data for analysis with *F*- and *R*- statistics (Hardy et al. 2003). The results of these tests showed that all of our 10 loci were better analyzed by *F*-statistics. The number of alleles (*N*_A_), expected and observed heterozygosities (*H*_E_, *H*_O_), and the inbreeding co-efficient (*F*_IS_) were calculated for each locus by site and globally for each locus using genepop v4 (Raymond and Rousset 1995).

We carried out tests of linkage disequilibrium for each locus by site using genepop v4. After Bonferroni correction (Rice 1989), significant departure from linkage disequilibrium was detected for 13 locus pairs across the sites (*P* < 0.01), although 12 of these pairs were found to depart from linkage disequilibrium at only one site. We followed recommendations by Kaeuffer et al. (2007) and estimated the correlation co-efficient (*r*_LD_; Black and Krafsur 1985) for these locus pairs using Linkdos software (Garnier-Gere and Dillman 1992) (http://genepop.curtin.edu.au/linkdos.html). The *r*_LD_ for each locus pair was <0.55 (*P* < 0.05), indicating a probable distance of greater than 3 cM between loci (Kaeuffer et al. 2007), which is sufficient distance that any linkage effect does not bias clustering analyses (Pritchard and Wen 2003); therefore, we retained all loci for further analyses. We tested for Hardy–Weinberg equilibrium within each site using genepop v4. After Bonferroni correction (Rice 1989), two loci, *Pn5* and *Pn15*, differed significantly from Hardy–Weinberg equilibrium at one and two sites respectively, both showing heterozygote deficiency (*P* < 0.01). We investigated the effect of these loci on our analyses by comparing results obtained with and without them. Results were consistent in all cases and we concluded that the observed departures from Hardy–Weinberg equilibrium for these loci would most likely not be strong enough to significantly bias results. Therefore, keeping these violations in mind, we retained all loci for further analyses, assuming that the extra information in these loci outweighs any potential biases they may add.

### Sex-biased dispersal

In birds with a resource defense mating system (Greenwood 1980; Clarke et al. 1997), such as *P. novaehollandiae* (McFarland 1985; Pyke et al. 1989), there is a tendency for male-biased philopatry, where males remain in or near their natal territory. Sex-biased dispersal can contribute to sexual variation in patterns of spatial (and temporal) genetic variation and phenology. We tested sex-biased dispersal using the program FSTAT v2.9.3.2 (Goudet 1995, 2001).

Goudet et al. (2002) recommend two tests with overlapping ranges of effectiveness for examining sex-biased dispersal – a test that examines sexual variation in the variance of assignment index (vAIc), and a test that examines sexual variation in the proportion of differential fixation of neutral alleles between samples (*F*_ST_). The vAIc test is ineffective at detecting biased dispersal when dispersal frequencies are high, but performs better than the *F*_ST_ test when dispersal frequencies are low. These tests are one-sided, based on the principle that genotypes of the more philopatric sex will be more similar (relative to the dispersing sex) in the population in which they were sampled. We tested the hypothesis that, in *P. novaehollandiae*, males are the more philopatric sex.

### Isolation by distance

Evidence from mark-recapture data of *P. novaehollandiae* indicates a limited capacity for dispersal (c.a. 110 km max.), shorter than the distances between some of our study sites (Paton et al. 2004). Museum vouchers and sight records (Fig. 2b), as well as a general survey of the land, suggest that, with the exception of the sea divides, there are no other obvious geographical or environmental discontinuities between our study sites that are likely to disrupt dispersal; therefore, we predict a pattern of isolation by distance. When dispersal is unrestricted in two dimensions, a positive regression slope of *F*_ST_/(1–*F*_ST_) on log of distance is expected (Rousset 1997), and we tested this. We used the program SPAGeDi v1.2 (Hardy and Vekemans 2002) to calculate pairwise *F*_ST_/(1–*F*_ST_) for each sex. We calculated pairwise distance measures as the shortest distance across land between two points (km), assuming dispersal between Kangaroo Island and mainland South Australia only across the smallest sea divide that separates the two (between the eastern tip of Kangaroo Island and the southern tip of Fleurieu Peninsula). Log of distance was subsequently computed from these distances. We evaluated the correlation between log of distance and pairwise *F*_ST_/(1−*F*_ST_) for each sex using 1 × 10^7^ randomizations. We accounted for non-independence of distance correlations inherent with matrix data by using Mantel tests (Mantel 1967; Mantel and Valand 1970) in the software program zt (Bonnet and Peer 2002).

### Genetic population structure analysis

To determine population structure using multi-locus genotype data, we used a Bayesian model-based clustering method in the program structure v2.3.2 (Pritchard et al. 2000; Falush et al. 2003, 2007; Hubisz et al. 2009). All Bayesian models assume (1) a prior distribution for unknown quantities (such as number of clusters); and (2) a likelihood function relating these unknown parameters to the observed genotypes. In structure, the unknown parameters are inferred through Markov Chain Monte Carlo (MCMC) computation. The number of clusters, *K*, is a fixed parameter set by the user – the model probabilistically assigns individuals to a cluster in a way that minimizes departure from Hardy–Weinberg equilibrium at each locus while conforming to the set value of *K*. The model assumes that loci *within* clusters are in Hardy–Weinberg equilibrium and linkage equilibrium. The procedure to find which value of *K* best fits the data consists of running multiple MCMC replicates for varying *K* and inferring the most likely value from the approximation of their posterior probabilities. Based on mark-recapture data of *P. novaehollandiae* (Paton et al. 2004), we expect structure to be relatively weak; therefore, we used the admixture model that allows individuals to have partial ancestry in each cluster – recommended for closely related populations (Pritchard et al. 2010). For the same reason, we used the option that takes into account the likelihood that allele frequencies are correlated across clusters. Pritchard et al. (2000) suggest that chains should converge within 1 × 10^4^ and 1 × 10^5^ MCMC iterations, so we investigated these properties by running multiple MCMC chains for different values of *K*. All chains converged with mixing within 3 × 10^4^ MCMC iterations; therefore, we chose a relatively conservative burn-in of 5 × 10^4^ MCMC iterations, which we fixed for all further runs. We tested sensitivity of the data to alternative hyper-parameter priors by running MCMC chains using different priors. Results were consistent between runs, but we reverted to the default priors (mean = 0.01, standard deviation = 0.05, lambda = 1) for all further runs as they make the algorithm more sensitive to subtle structure (Falush et al. 2003). Exploration of the data for consistency across longer and shorter chains for a range of *K* indicated that a chain length of 1 × 10^5^ MCMC iterations was most appropriate. Using our optimized burn-in length (5 × 10^4^ iterations) and MCMC length (1 × 10^5^ iterations), we ran 10 MCMC replicates for *K* = 1−8. Although there is some debate about the best method for inferring clusters (Evanno et al. 2005), our data appear to best fit the method published in the original structure paper (we expect homogeneous patterns of dispersal between clusters), which involves comparing mean log likelihoods penalized by one-half of their variance (Pritchard et al. 2000).

### Phenotypic and genetic variation *(P*_ST_ *and F*_ST_*)*

We examined the role of genetic drift as a possible process for a pattern of phenotypic divergence. The influence of genetic drift on variation in a phenotypic trait can be estimated by comparing neutral genetic variation with phenotypic variation between populations. If phenotypic variation significantly exceeds neutral genetic variation, there is evidence to suggest that the trait has diverged more than expected by genetic drift alone (Leinonen et al. 2006; Raeymaekers et al. 2007).

As a measure of neutral genetic variation, we estimated the differential fixation of neutral alleles, *F*_ST_ (Weir and Cockerham 1984), calculated using the program SPAGeDi v1.2 (Hardy and Vekemans 2002). To estimate the variance in *F*_ST_, we estimated 95% confidence intervals for *F*_ST_ by jack-knifing over loci. As a measure of phenotypic variation, we used *P*_ST_ (Leinonen et al. 2006; Raeymaekers et al. 2007), a phenotypic analog of *F*_ST_ derived from a measure of quantitative genetic differentiation, *Q*_ST_ (Spitze 1993). There is much discussion over the suitability of comparing *Q*_ST_ and associated measures with *F*_ST_ for inferring the role of natural selection (O'Hara and Merilä 2005; Pujol et al. 2008; Whitlock 2008; Edelaar and Björklund 2011), but we avoid much of this controversy by restricting our investigation to inference of the role of genetic drift only. *P*_ST_ calculates the within- and among-population variance components of phenotypic variance such that:


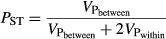
(1)

where 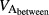 and 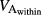 are the phenotypic variances between and within populations. We calculated the morphological variances of each trait among pairwise populations by performing a one-way analysis of variance (ANOVA) using population as the dependent variable, for each sex. We used the mean square estimates to calculate the between- (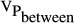) and within- (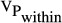) population variance. We calculated *P*_ST_ between all pairwise comparisons for each morphology variable for each sex. Conventional methods for estimating variance in *P*_ST_ estimates consider a global *P*_ST_ and necessitate a large number of populations (Meirmans and Hedrick 2011), two conditions not met by our data. Therefore, as a conservative measure, we calculated the standard deviation of *P*_ST_ estimates across pairwise comparisons and used them as upper and lower confidence intervals.

Due to the nature of the summary statistics *F*_ST_ and *P*_ST_, both may suffer from sampling bias (discussed in Meirmans and Hedrick 2011); however, as *P*_ST_ is based on *F*_ST_, any biases are likely to be shared. Therefore, subsequent correction for sampling bias should not be required on these statistics, especially considering that microsatellite data have been used to estimate *F*_ST_ (Merilä and Crnokrak 2001).

## Results

### Morphology and rainfall

Morphological trait values in both males and females differed significantly across study sites (MANOVA; Male: *F* = 3.61; *P* < 0.001; Wilk's Lambda = 0.73; Partial ETA^2^ = 0.06; Female: Male: *F* = 1.83; *P* = 0.005; Wilk's Lambda = 0.77; Partial ETA^2^ = 0.05) (see Table 1). In males, all traits except bill width differed significantly across study sites after Bonferroni adjustment (Table 2); in females, only bill depth differed significantly across study sites, but bill-head length, bill-nostril length, and bill width showed near-significant variation (Table 2). We used Multiple Regression Analysis to test if rainfall was associated with the observed patterns of morphological variation. In both males and females, mean annual rainfall per study site correlated with morphology in accordance with our predictions (Table 3). Birds had longer bill-head length (but not bill-nostril length), shallower bills, and shorter tarsi in higher rainfall areas.

**Table 2:** Male and female New Holland Honeyeater morphology in South Australia differs significantly across study sites at a landscape scale (refer to Methods). Results are shown for ANOVA; shaded variables were significant at *P* < 0.05 after sequential Bonferroni adjustment. The corrected total was 357 males and 223 females

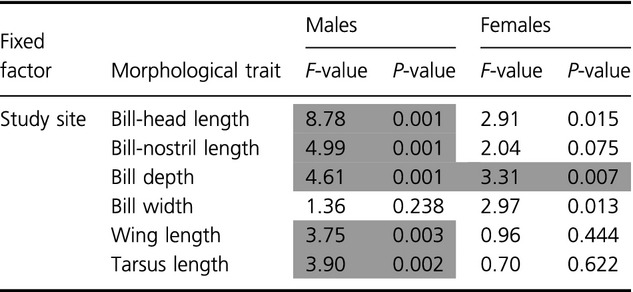

**Table 3:** The relationship between morphology and rainfall per study site. Results are shown for multiple regression analysis with mean annual rainfall (dependent variable) against the morphological traits (independent variables). Shaded variables indicate significance at *P* < 0.05 after sequential Bonferroni adjustment. In areas with high rainfall, birds had long and shallow bills and short tarsus length

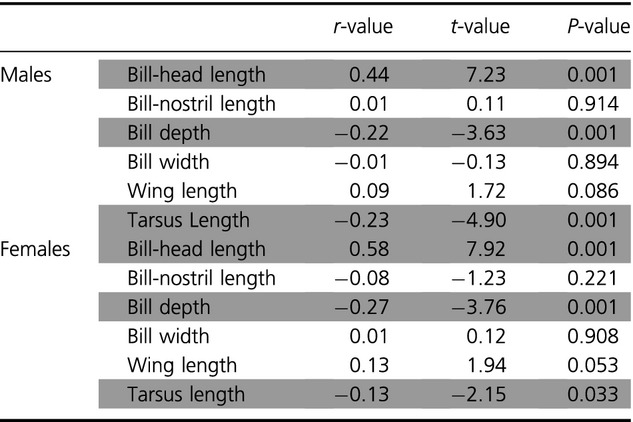

### Body condition in relation to morphology and rainfall

**Figure 3:**
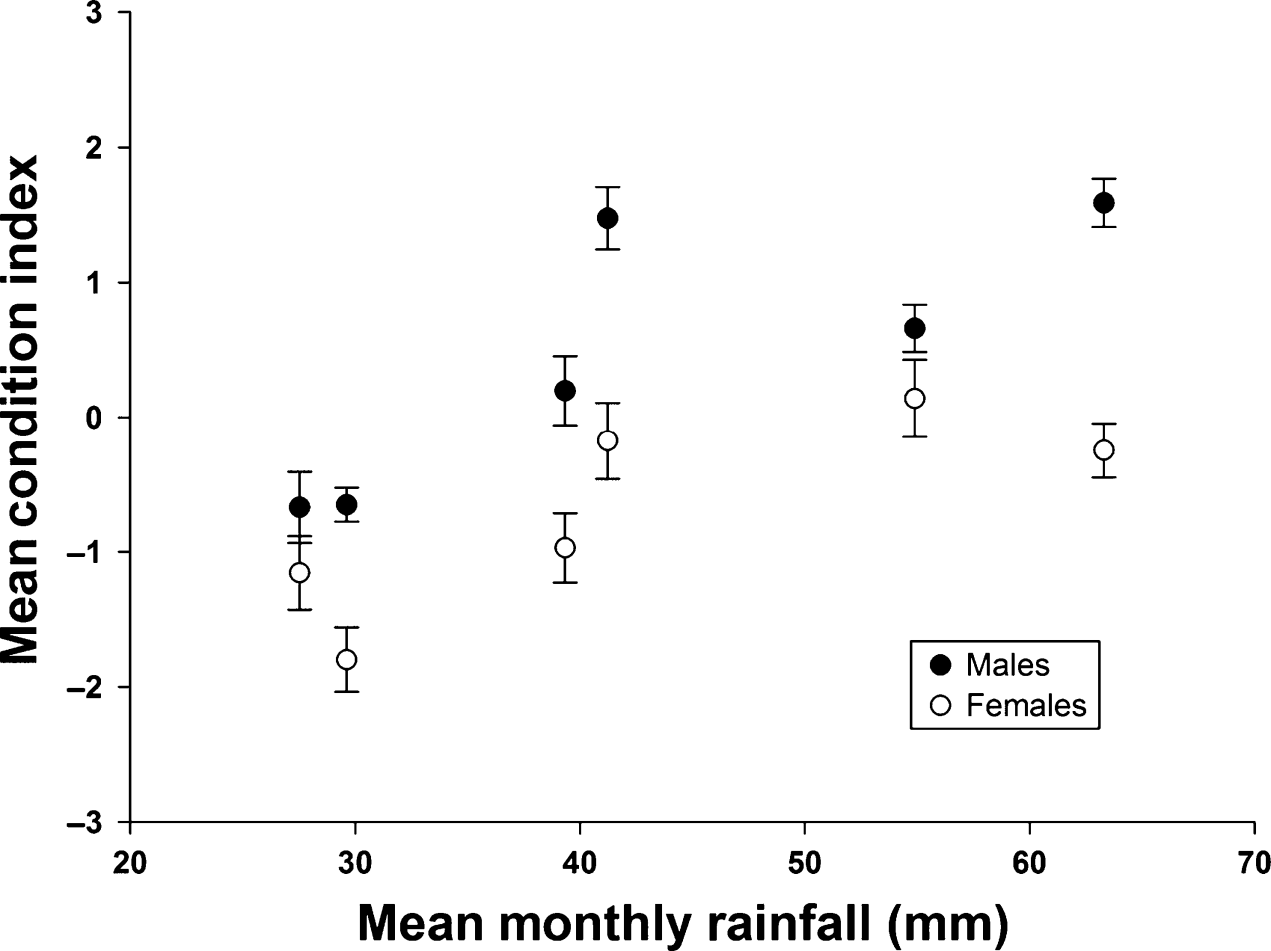
Mean body condition (mean ± se) plotted in relation to the mean monthly rainfall for each study site in male and female New Holland Honeyeaters in South Australia.

To test the assumption that body condition is higher in areas of high rainfall, we compared condition index and rainfall across our study sites. We found a positive correlation between condition index and mean annual rainfall per study site in males (adjusted r^2^ = 0.30, *P* < 0.001) and females (*r* = 0.15, *P* = 0.031): birds in areas with higher rainfall had higher mean condition index values (Fig. 3).

**Figure 4:**
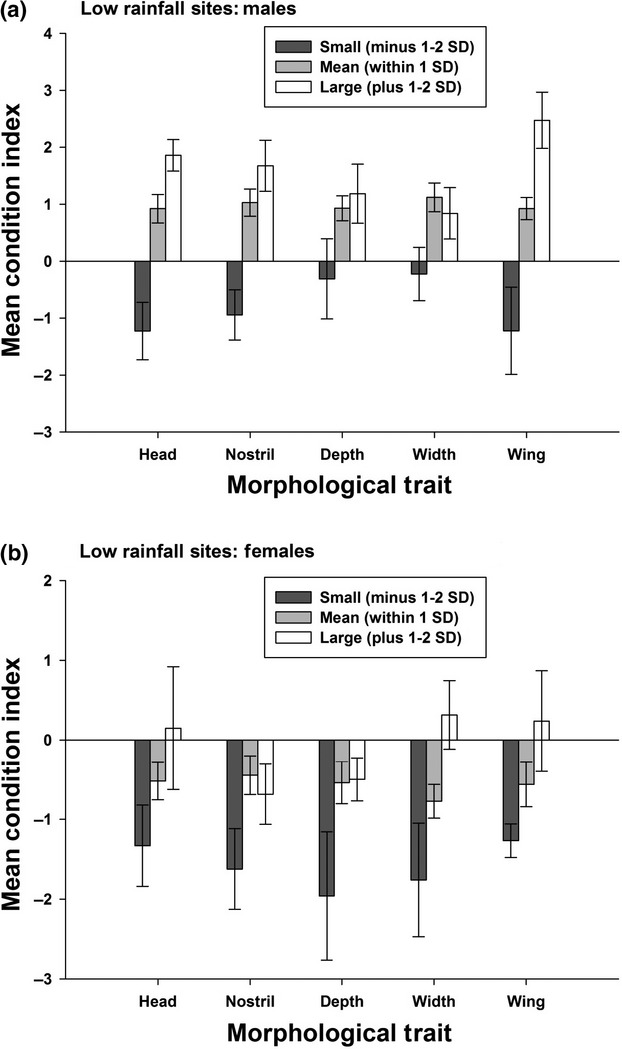
Condition index (mean ± se) in birds in low-rainfall areas. We compare mean body condition index in relation to morphological trait values within three size categories per trait: small (between 1 and 2 SD smaller than the mean trait value), mean (within 1 SD of the mean), and large between 1 and 2 SD larger than the mean trait value) in (a) males and (b) females. The morphology variables were bill-head length (head), bill-nostril length (nostril), bill depth (depth), bill width (width), and wing length (wing).

**Figure 5:**
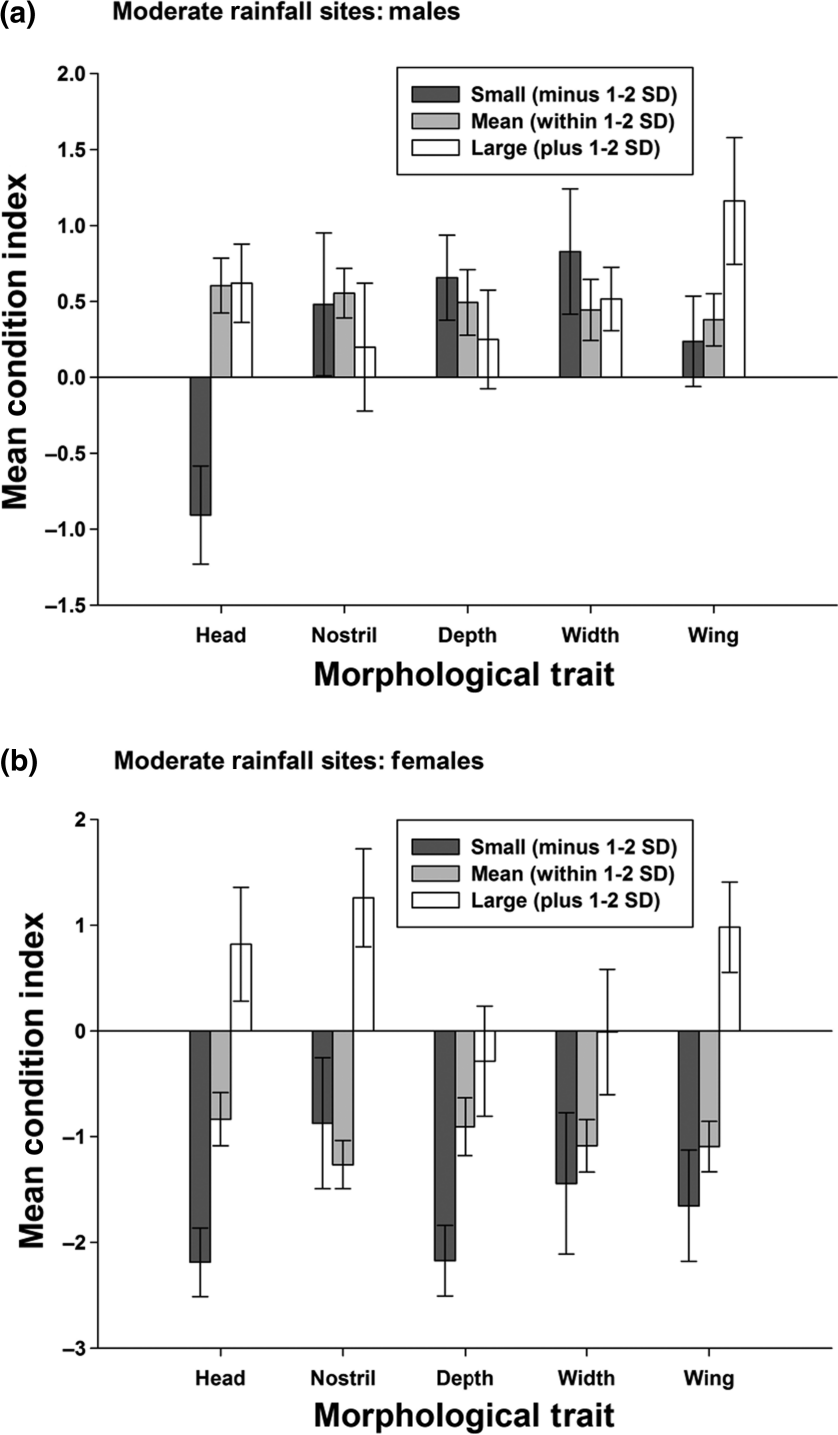
Condition index (mean ± se) in birds in moderate-rainfall areas. We compare mean body condition index in relation to morphological trait values within three size categories per trait: small (between 1 and 2 SD smaller than the mean trait value), mean (within 1 SD of the mean), and large (between 1 and 2 SD larger than the mean trait value) in (a) males and (b) females. The morphology variables were bill-head length (head), bill-nostril length (nostril), bill depth (depth), bill width (width), and wing length (wing).

**Figure 6:**
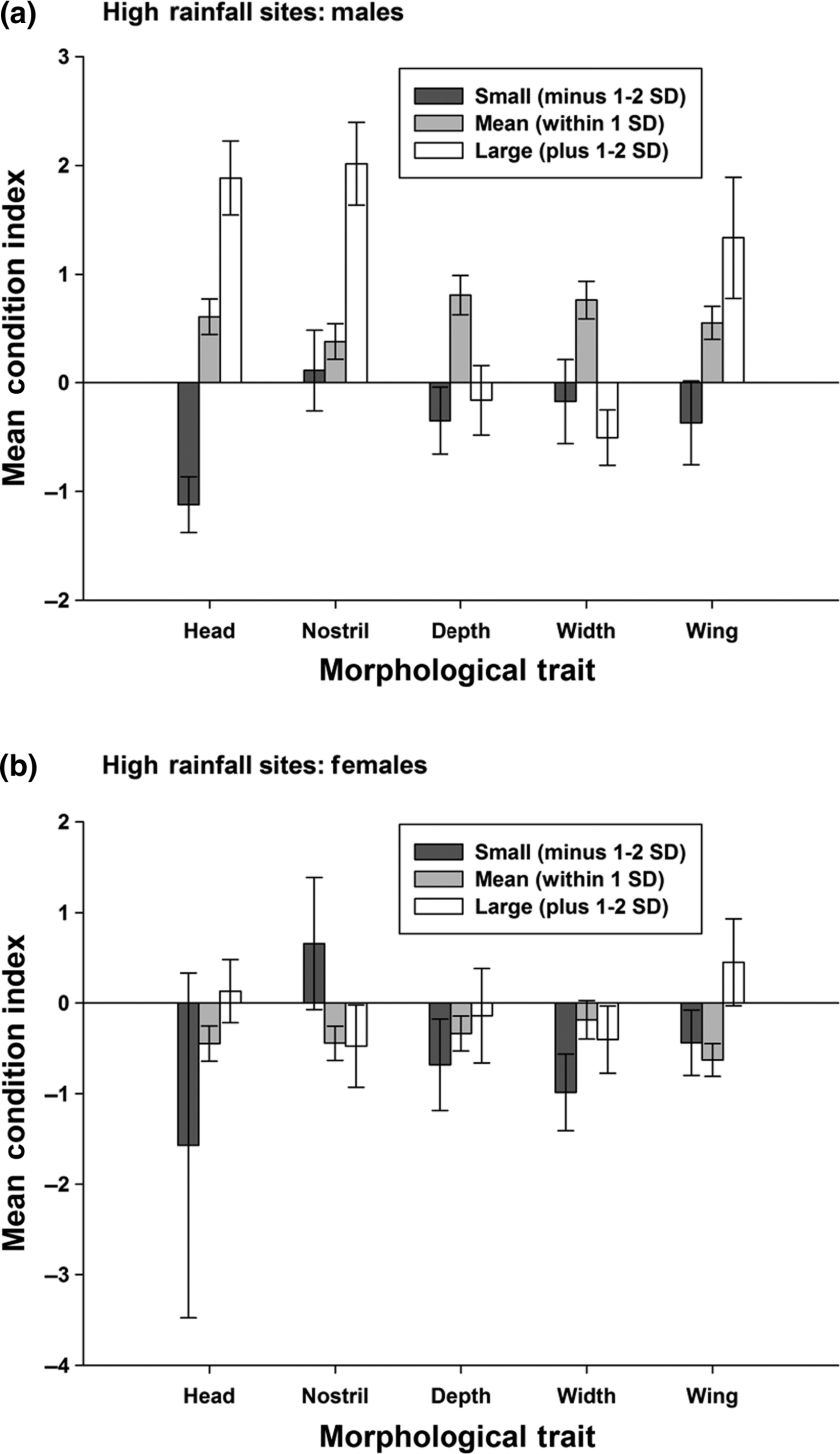
Condition index (mean ± se) in birds in high-rainfall areas. We compare mean body condition in relation to morphological trait values within three size categories per trait: small (between 1 and 2 SD smaller than the mean trait value), mean (within 1 SD of the mean), and large (between 1 and 2 SD larger than the mean trait value) in (a) males and (b) females. The morphology variables were bill-head length (head), bill-nostril length (nostril), bill depth (depth), bill width (width), and wing length (wing).

To test for possible adaptive explanations for the relationships observed between morphology and rainfall, we compared condition index, an indirect proxy for fitness, with morphology, using One-Way Between-Groups Analysis of Variance. The pattern in males and females was noticeably different across rainfall categories (Table 4). The relationships observed between condition index and morphology that can be used to identify the mode and direction of selection are shown in Figs 4–6. Here, we report those relationships that were shown to be significant. In low-rainfall sites, males with longer bill-head length, bill-nostril length, and wing length, had higher condition indices. In moderate-rainfall sites, females with longer bill-head length, bill-nostril length, and wing length, had higher condition indices. In high-rainfall sites, males with mean trait values for bill width and bill depth, and with longer bill-head length, bill-nostril length, and wing length, had higher condition indices.

**Table 4:** Variation in mean condition index across morphological size categories, within each rainfall category (low, moderate, high), for each sex. Results are shown for one-way between-groups analysis of variance. Shaded variables indicate significance at *P* < 0.05 after sequential Bonferroni adjustment. The relationship between body condition and morphological trait value was similar in males and females within each rainfall category

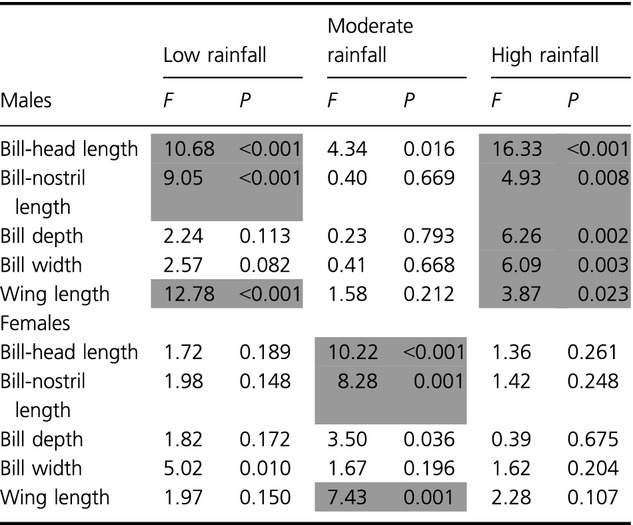

### Molecular genetic analysis

Of 3300 data points (genotypes for 330 individuals at 10 loci), there were 192 (5.8%) missing values. The number of alleles per locus ranged from 6 to 18 (mean 13.5), expected heterozygosity ranged from 0.67 to 0.91 (mean 0.83), and allelic richness ranged from 4.45 to 14.83 (mean 10.15).

### Sex-biased dispersal

We found evidence for male-biased philopatry: male vAIc was not significantly greater than female vAIc within sites (*P* = 0.081), but male *F*_ST_ was significantly lower than female *F*_ST_ within sites (*P* = 0.038). Comparing our results with those of exhaustive sampling by Goudet et al. (2002), we can estimate the dispersal rate (the proportion of juvenile individuals moving from one study site to another) in *P. novaehollandiae* to be approximately 20% greater in females than in males. Considering that males in our study significantly outnumbered females (61.9% of 635 birds were males, *χ*^*2*^ = 30.43, *P* < 0.001), then the dispersal rate in males should be no more than 53%.

### Isolation by distance

Mantel tests examining the correlation between pairwise *F*_ST_/(1 − *F*_ST_) and log of pairwise distance showed significant positive correlations in both males (*r* = 0.77, *P* = 0.033) and females (*r* = 0.75, *P* = 0.024), with a substantial proportion (>75%) of the neutral genetic variation between sites being explained by distance between sites.

### Genetic population structure analysis

We found a single continuous genetic population at a landscape scale. Estimates of the logarithm of probability of the data averaged over the 10 MCMC replicates for each value of *K* were maximal for *K* = 1 under the standard admixture model. As population differentiation appears to be low for these data, we also implemented the locprior model – a structure model that incorporates site information in the inference by using a modified prior distribution for clustering that allows the distribution of cluster assignments to vary by site (Hubisz et al. 2009). Applying the locprior model (that assumes admixture) to our data, allowing for correlated allele frequencies, we investigated convergence and mixing properties of chains, and sensitivity of the data to alternative hyper-parameter priors, as previously described in the Methods for the standard model. All chains converged with mixing within 2 × 10^4^ MCMC iterations and results were consistent between runs; therefore, we chose a relatively conservative burn-in of 5 × 10^4^ MCMC iterations, which we fixed for all further runs, and we reverted to the default priors for all runs (explained in Materials and Methods). Exploration of the data for consistency between replicate runs indicated a chain length of 1 × 10^5^ MCMC iterations was most appropriate. Using our optimized burn-in length (5 × 10^4^ iterations) and MCMC length (1 × 10^5^ iterations), we ran 10 MCMC replicates for *K* = 1–8. Averaged over the 10 MCMC replicates for each value of *K*, the logarithm of probability of the data was maximal for *K* = 3, different from the estimate of *K* = 1 made using the standard model. The three clusters generally represented spatial groups, with individuals from sites within close proximity clustering together; most individuals from “Low Rainfall 2” clustered together, as did most individuals from “Low Rainfall 1,” while the remaining individuals formed a third cluster. Two facts support the findings of the locprior model over those of the standard model: (1) the clusters make biological sense by conforming to patterns of geographic isolation; and (2) cluster membership indicates shared ancestry between clusters, expected under high gene flow. Therefore, population estimates based on the locprior model should most accurately represent the true populations. We believe, the most likely reason for the standard structure model failing to detect structure was the strong signal of isolation by distance, which can swamp clustering estimates (Pritchard and Wen 2003).

We carried out standard molecular genetic analysis for microsatellite loci within the three clusters identified by structure. The number of alleles (*N*_A_), expected and observed heterozygosities (*H*_E_, *H*_O_), and the inbreeding co-efficient (*F*_IS_) were calculated for each locus by cluster using genepop v4. We carried out tests of linkage disequilibrium for each locus by cluster; after Bonferroni correction, significant departure from linkage disequilibrium was detected for one locus pair in the largest cluster (*P* < 0.01). The estimated correlation co-efficient (*r*_LD_) for this locus pair, calculated using Linkdos, indicated that the loci were most likely separated by a distance of greater than 3 cM (*r*_LD_ <0.55; *P* < 0.05). We tested for Hardy–Weinberg equilibrium within each cluster; after Bonferroni correction, two loci, *Pn4* and *Pn5*, differed significantly from Hardy–Weinberg equilibrium in the largest cluster, both showing heterozygote deficiency (*P* < 0.01).

### Phenotypic and genetic variation *(P*_ST_ *and F*_ST_*)*

In males, *F*_ST_ among sites ranged from 0.002 to 0.047. Eight of 15 pairwise comparisons were significantly different from 0 (Table 5). In females, *F*_ST_ among sites ranged from 0.000 to 0.032. Two pairwise comparisons showed negative *F*_ST_ (effectively 0) and one showed an *F*_ST_ of 0. Six pairwise comparisons were significantly different from 0 (Table 5). A geographic pattern was observed for *F*_ST_ in males and females: all significant pairwise comparisons included either “Low Rainfall 1” or “Low Rainfall 2,” the two most isolated sites. Phenotypic variation (*P*_ST_) exceeded neutral genetic variation (*F*_ST_) for all tested morphological variables in both males and females.

**Table 5:** Variation in *F*_ST_ for pairwise comparisons of sites for males (top matrix) and females (side matrix). *P*-values were obtained after 21,000 permutations and significant deviations above 0 (*P* < 0.003 after adjustment for multiple comparisons) are shaded

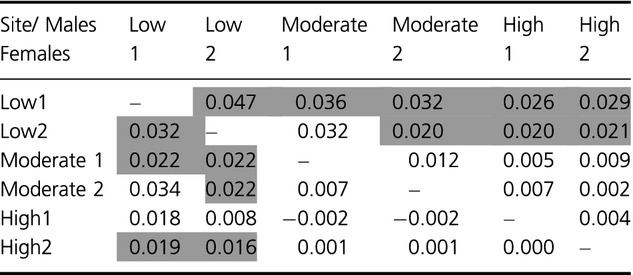

## Discussion

This study found a single continuous genetic population structure at a landscape scale in a key pollinator species, *P. novaehollandiae*, across one island and three peninsulas in South Australia. There was sex-biased dispersal: males were the philopatric sex and females, the dispersing sex. Finally, despite high gene flow, we found phenotypic differences between sites, which correlated with rainfall as predicted. Birds in low-rainfall areas had shorter bill-head length, deeper bills, longer tarsi, and lower body condition. Despite these relationships, a signal of stabilizing selection was detected only in bill depth, and only in males in high-rainfall sites.

The pattern of isolation by distance (IBD) observed in both sexes was particularly strong (*r* ≥ 0.75), which we expected with the relative absence of physical barriers (after accounting for the sea divides) coupled with the limited dispersal capacity of *P. novaehollandiae* across the study area (Higgins and Peter 2002). A signal of IBD confirms that gene flow between populations is possible, but restricted in a predominantly distance-related sense (Slatkin 1993; Rousset 1997; Hutchinson and Templeton 1999). The geographic pattern that we found for pairwise estimates of neutral allelic fixation (*F*_ST_) is consistent with the observed pattern of IBD. Under IBD, allele frequencies are expected to vary gradually with distance, and genotypes from the most remote sites are expected to be most differentiated. Accordingly, we found that pairwise comparisons including either “Low Rainfall 1” or “Low Rainfall 2,” our two most remote sites, were the only sites with significant *F*_ST_, which is also reflected in the number and composition of clusters found by structure using the more powerful locprior model. The contrasting finding from the standard structure model and the locprior model probably reflects insensitivity of structure to an underlying population model of IBD with inadequate spatial sampling of populations in a “stepping stone” distribution (Pritchard et al. 2010).

On the basis of expected ecological function of the morphological traits under investigation, and their interaction with the environment, we predicted that variation in morphology between sites that could be explained by rainfall should exceed neutral genetic variation. In all cases, estimates of phenotypic variation, *P*_ST_, exceeded those of neutral genetic variation, *F*_ST_. However, in estimating *F*_ST_, two microsatellite loci, *Pn4* and *Pn5*, showed significant heterozygote deficit, probably caused by null alleles. Genetic distances and *F*_ST_ tend to be overestimated in the presence of null alleles (Chapuis and Etoup 2007); however, as our *P*_ST_ estimates always exceeded *F*_ST_ estimates and our *F*_ST_ estimates were uniformly low, null alleles should not affect interpretation of our results. To test the effect of the loci *Pn4* and *Pn5* on our *F*_ST_ estimates, we re-calculated the global *F*_ST_ using the same data set, but with these loci removed – the subsequent *F*_ST_ estimates (male = 0.016; female = 0.008) were lower than the original estimates (male = 0.018; female = 0.013), as expected. Subsequently, the large values that we observed for *P*_ST_ for all morphological variables, relative to *F*_ST_ in males and females, indicate a negligible influence of genetic drift in the divergence of morphological traits of *P. novaehollandiae* in our study area (Leinonen et al. 2006; Raeymaekers et al. 2007).

We predicted that in areas of low rainfall, there should be reduced availability of nectar – the primary food source of *P. novaehollandiae* – which should drive niche expansion via increased dependence on insects, an important supplementary food source (see Myers et al. 2010). Therefore, we predicted that birds in low-rainfall areas would have deeper bills – an adaptation to a more insectivorous diet, as a deeper bill provides increased crushing force, and bill depth is known to correlate with increased range of prey size for consumption by birds (Bowman 1961; Lederer 1975; Grant 1999). We found some evidence for this pattern. Birds in low-rainfall sites had lower condition indices. Also, bill depth in males and females differed across sites and correlated with rainfall as expected based on ecological function. However, a signal of stabilizing selection – an indication of local adaptation – was only observed for bill depth in males in high-rainfall sites. The absence of local selective signals for bill depth in females, and in males in low- and moderate-rainfall sites, may be due to interference from other sources of selection, such as a source of strong global selection. This idea is explored in more detail below. The signal of stabilizing selection that we observed in males could be explained by density-dependent selection: in a resource defense mating system where males defend territories, such as that of *P. novaehollandiae* (McFarland 1985; Pyke et al. 1989), male–male competition is expected to increase in larger populations as the frequency of intruders increases (McFarland 1986, 1996; Armstrong 1991b); therefore, if favorable conditions in high-rainfall sites stimulate population growth, then increased male – male competition may amplify the strength of selection on important ecological traits, such as bill depth, at these sites. Either way, our findings support the hypothesis that local selective forces, influenced by rainfall, have driven divergent natural selection in morphology of an ecological trait across the climatically variable landscape of South Australia.

In addition to evidence for selection acting at a local scale, we found evidence for a global pattern of selection (inferred from the distribution of condition indices in relation to morphology). The mode and direction of selection in each trait for males and females were similar. With the exception of bill depth and width, selection was always directional toward larger size. Considering the broad scale of this pattern, it is unlikely that it is predominantly explained by local conditions, but rather is shaped by a large-scale mechanism, such as long-term global climate change. During the study period, the climate in South Australia was relatively dry (http://www.bom.gov.au/climate/current/statements/scs9a.pdf). Under drier conditions, less nectar is expected to drive a subsequent shift toward insectivory in *P. novaehollandiae*. This shift in foraging is predicted to be followed by adaptive morphological change toward shorter but deeper bills, and longer wings. While there appears to be directional selection in favor of longer winged birds in our study area, longer billed birds also appear to be favored. While a shorter, deeper bill provides increased crushing force and access to a greater diversity of prey (Bowman 1961; Lederer 1975; Grant 1999), a longer bill is generally more efficient at catching fast moving prey, (Lederer 1975) such as the small aerial insects most commonly targeted by *P. novaehollandiae* (Paton 1982). Contrary to what we predicted for drier conditions, this observation suggests that an increase in foraging efficiency – not foraging diversity – may be favored in *P. novaehollandiae*, perhaps driven by resource competition with more insectivorous birds that already populate the area. It is worth noting that the observation of longer bills in drier conditions is also contrary to what would be expected under adaptive morphological change influenced by the alternate carbohydrate sources of *P. novaehollandiae*, manna and lerp. If an adaptive mechanism is responsible for the observed pattern, and selection pressure – reflected by increased mortality – is greatest under dry conditions (*sensu* Mac Nalley et al. 2009), as our observations suggest, then the further aridification that is predicted for South Australia (Klausmeyer and Shaw 2009) may lead to future population declines.

On the basis of combined evidence of our data, we conclude that rainfall has shaped aspects of phenology in *P. novaehollandiae* in South Australia, both locally and globally, with a consistent pattern of adaptive divergence at a landscape scale. The conclusions of this study hinge largely upon the assumption that nectar availability is directly correlated with rainfall. Although we provided support for this assumption from the literature (Porter 1978; Armstrong 1991a; Wyatt et al. 1992; Wooller et al. 1998; Keasar et al. 2008; Watson 2011), we did not test the assumption directly. A quantification of nectar availability in relation to rainfall, and in relation to *P. novaehollandiae* diet, would test some of the arguments presented in this study. Despite these shortcomings, this study provides important baseline data about a key pollinator system in South Australia. For example, this is the first study to show high gene flow at a landscape scale in *P. novaehollandiae*, and to provide genetic evidence for sex-biased dispersal in the focal species. In addition, we have provided some evidence for the adaptive capacity of this key pollinator species. Notwithstanding this indirect evidence for adaptive shifts in phenotype, *P. novaehollandiae* is declining in parts of South Australia (Szabo et al. 2011), which may indicate a limited capacity for adaptive responses to changing environments, with important implications for conservation (see also Paton 2000; Higgins and Peter 2002; Mac Nalley et al. 2009; Ford 2011; Sunnucks 2011).
